# Simple BiCMOS CCCTA Design and Resistorless Analog Function Realization

**DOI:** 10.1155/2014/423979

**Published:** 2014-07-16

**Authors:** Worapong Tangsrirat

**Affiliations:** Faculty of Engineering, King Mongkut's Institute of Technology Ladkrabang (KMITL), Ladkrabang, Bangkok 10520, Thailand

## Abstract

The simple realization of the current-controlled conveyor transconductance amplifier (CCCTA) in BiCMOS technology is introduced. The proposed BiCMOS CCCTA realization is based on the use of differential pair and basic current mirror, which results in simple structure. Its characteristics, that is, parasitic resistance (*R*_*x*_) and current transfer (*i*_*o*_/*i*_*z*_), are also tunable electronically by external bias currents. The realized circuit is suitable for fabrication using standard 0.35 *μ*m BiCMOS technology. Some simple and compact resistorless applications employing the proposed CCCTA as active elements are also suggested, which show that their circuit characteristics with electronic controllability are obtained. PSPICE simulation results demonstrating the circuit behaviors and confirming the theoretical analysis are performed.

## 1. Introduction

Since an introduction of the newly defined active building block, namely, the current conveyor transconductance amplifier (CCTA), in 2005 [1], this device has been gaining an increasing attention that led to a great number of analog function circuits. Basically, the CCTA device can be realized by cascading the second-generation current conveyor with the multioutput transconductance amplifier in monolithic form. By combining the advantages of both circuit technologies, the CCTA possesses low power consumption, wide bandwidth, high dynamic range, and high-slew rate. Considering these reasons, the CCTA is suitable for a class of analog signal processing which can process both current and voltage signals. Hence, a great number of numerous analog adjustable functions are available in open literature [1–5].

In 2008, the current-controlled conveyor transconductance amplifier (CCCTA), which is a modified version of the CCTA, was introduced in bipolar technology [6]. Since its introduction, the CCCTA has been widely used in applications for continuous-time signal processing [6–9]. The parasitic resistance looking into the *x*-terminal (*R*_*x*_) of the circuit is used to advantage in current-controlled circuit parameter, because it is easily adjusted by an external biasing current. This advantage allows the implementation of numerous electronically tunable circuits without requiring external passive resistors, which is especially important for integrated circuit implementation.

In recent integrated circuit technology, there are two basic technologies that are known as bipolar and CMOS technologies. For the bipolar transistor technology, they have higher transconductance gain (*g*_*m*_), low-noise performance, and better high-frequency performance than their CMOS counterparts [10, 11]. On the other hand, the advantage of the CMOS technology includes high-input impedance level, low power dissipation, and small chip area. The circuit realized in BiCMOS technology will therefore provide the advantages of both technologies.

The aim of this work is to realize a current-controlled conveyor transconductance amplifier (CCCTA) structure suitable for integration in BiCMOS technology. The proposed CCCTA has relatively simple structure, since it is composed of solely differential pairs and simple current mirrors. The important circuit parameters, that is, parasitic resistance *R*_*x*_ and current transfer characteristic *i*_*o*_/*i*_*z*_, can be adjusted electronically and linearly through the external bias currents. The characteristics of the proposed CCCTA are demonstrated by PSPICE simulation results using 0.35 *μ*m BiCMOS real process parameters. The results show good agreement with the expected values. Some application examples in realizing resistorless analog function circuits using the proposed CCCTA as active building blocks are also given. The circuit solutions with fewer components are realized in order to demonstrate the easy applicability of the proposed circuit and to obtain simple and compact circuit designs.

## 2. Basic Concept of the CCCTA

**Figure 1:**
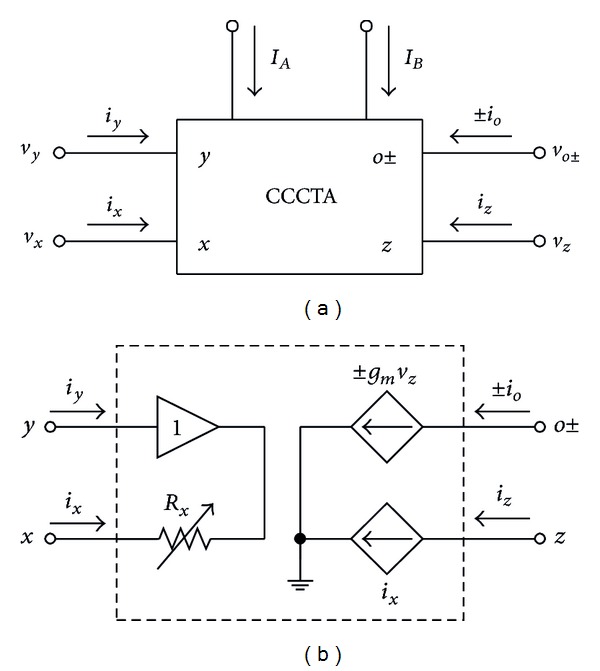
The CCCTA: (a) circuit symbol and (b) its equivalent circuit.

The CCCTA is conceptually a combination of second-generation current-controlled conveyor (CCCII) and transconductance amplifier. Its electrical symbol and equivalent circuit can be shown in Figure 1. As shown, the CCCTA device consists of two input terminals (*y* and *x*) and two output terminals (*z* and *o*). The *x*-terminal has a parasitic serial resistance (*R*_*x*_), where its value usually depends on an external supplied current. The *y*-terminal is the high-input impedance terminal, while the *z*- and *o*-terminals are two types of high-output impedance terminals. The property of the CCCTA can be described by the following matrix:

(1) $$\begin{array}{c} \left[\begin{array}{c} i_{y}\\ v_{x}\\ i_{z}\\ i_{o\pm } \end{array}\right]=\left[\begin{array}{cccc} 0 & 0 & 0 & 0\\ R_{x} & 1 & 0 & 0\\ 1 & 0 & 0 & 0\\ 0 & 0 & \pm g_{m} & 0 \end{array}\right]\cdot \left[\begin{array}{c} i_{x}\\ v_{y}\\ v_{z}\\ v_{o\pm } \end{array}\right], \end{array}$$

where *R*_*x*_ and *g*_*m*_ are the finite parasitic resistance looking into the *x*-terminal and the transconductance gain of the CCCTA, respectively. Here, *R*_*x*_ and *g*_*m*_ depend on the external DC bias currents *I*_*A*_ and *I*_*B*_, respectively.

## 3. Proposed BiCMOS CCCTA Realization

**Figure 2:**
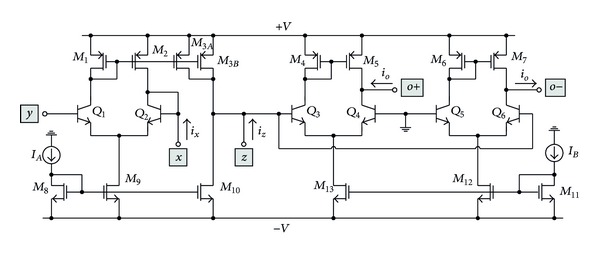
BiCMOS realization of the proposed CCCTA.

The schematic BiCMOS realization of the proposed CCCTA is shown in Figure 2. The circuit mainly consists of second-generation current-controlled conveyor (CCCII) and transconductance amplifier. It is designed by combining bipolar and CMOS technologies in order to utilize the main advantages of each technology, that is, higher transconductance, higher frequency, low power consumption, and small silicon area. The groups of transistors *Q*_1_-*Q*_2_, *Q*_3_-*Q*_4_, and *Q*_5_-*Q*_6_, which are assumed to be well matched, act as transconductance amplifiers to convert the voltage signal to the current signal. The current mirroring has been achieved by simple current mirror circuits (*M*_1_–*M*_3_), (*M*_4_-*M*_5_), (*M*_6_-*M*_7_), (*M*_8_–*M*_10_), and (*M*_11_–*M*_13_). Mirroring actions between *M*_1_ and *M*_2_, *M*_4_ and *M*_5_, and *M*_6_ and *M*_7_ force equal bias current in *Q*_1_ and *Q*_2_, *Q*_3_ and *Q*_4_, and *Q*_5_ and *Q*_6_, respectively. By applying the translinear principle to the base-emitter voltages (*v*_BE_) of *Q*_1_ and *Q*_2_, the differential input voltage (*v*_*y*_ − *v*_*x*_) can be derived as

(2) $$\begin{array}{c} v_{y}-v_{x}=v_{\text{BE}1}-v_{\text{BE}2}=V_{T}\left(\ln\frac{i_{c1}}{I_{S}}-\ln\frac{i_{c2}}{I_{S}}\right), \end{array}$$

where *V*_*T*_≅26 mV at 27°C is the thermal voltage, *I*_*S*_ is the reverse saturation current, and *i*_*c*1_ and *i*_*c*2_ are, respectively, the collector currents of transistors *Q*_1_ and *Q*_2_.

As shown in Figure 2, the relationship of the current flowing through the *x*-terminal (*i*_*x*_) is equal to

(3) $$\begin{array}{c} i_{x}=i_{c2}-i_{c1}, \end{array}$$

where

(4) $$\begin{array}{c} i_{c1}=\frac{I_{A}}{1+e^{-(v_{y}-v_{x})/V_{T}}},\\ i_{c2}=\frac{I_{A}}{1+e^{(v_{y}-v_{x})/V_{T}}}. \end{array}$$

Substituting (4) into (3), the current *i*_*x*_ can be rewritten as

(5) $$\begin{array}{c} i_{x}=I_{A}\tanh\left(\frac{v_{x}-v_{y}}{2V_{T}}\right). \end{array}$$

For simplification, if we assume that (*v*_*x*_ − *v*_*y*_) ≪ 2*V*_*T*_, then the term tanh(*v*_*x*_ − *v*_*y*_/2*V*_*T*_) can be approximately reduced to (*v*_*x*_ − *v*_*y*_/2*V*_*T*_). Hence, (5) can also be given by

(6) ix≅IA(vx−vy2VT).

From the above expression, the parasitic resistance looking into the *x*-terminal (*R*_*x*_) of the CCCTA when the *y*-terminal is connected to ground has been derived as

(7) $$\begin{array}{c} R_{x}≅\frac{2V_{T}}{I_{A}}. \end{array}$$

It should be noted from (7) that the resistance *R*_*x*_ is controllable electronically by adjusting the bias current *I*_*A*_.

Similarly, the small-signal transconductance gain (*g*_*m*_) of the CCCTA derived from transconductors *Q*_3_-*Q*_4_ (*Q*_5_-*Q*_6_) can be expressed as [12]

(8) $$\begin{array}{c} g_{m}=\frac{i_{o}}{v_{z}}=\frac{I_{B}}{2V_{T}}. \end{array}$$

Also note that the *g*_*m*_-value can be controlled electronically and linearly by changing the *I*_*B*_-value.

## 4. Simulation Results

To confirm the theoretical study, the proposed CCCTA structure in Figure 2 has been simulated with PSPICE using standard 0.35 *μ*m BiCMOS process parameters. The circuit was biased with ±1 V supply voltages. The transistor aspect ratios (*W*/*L* in *μ*m/*μ*m) were chosen as 7/0.7 and 8.5/0.7 for all the PMOS and NMOS transistors, respectively.

**Figure 3:**
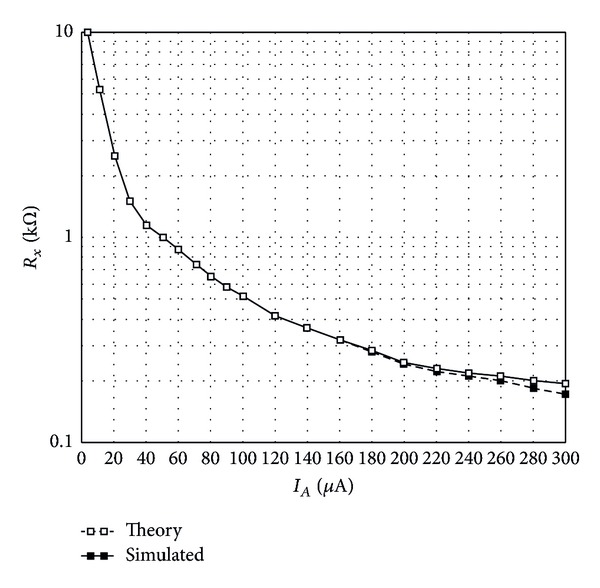
Variation of *R*_*x*_ as a function of *I*_*A*_.

**Figure 4:**
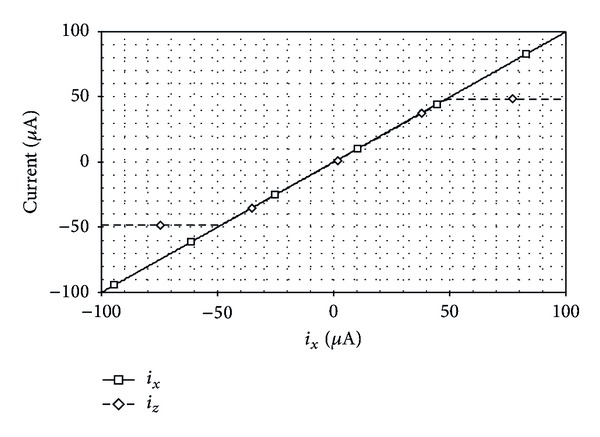
Simulated DC current transfer characteristic between *i*_*x*_ and *i*_*z*_.

**Figure 5:**
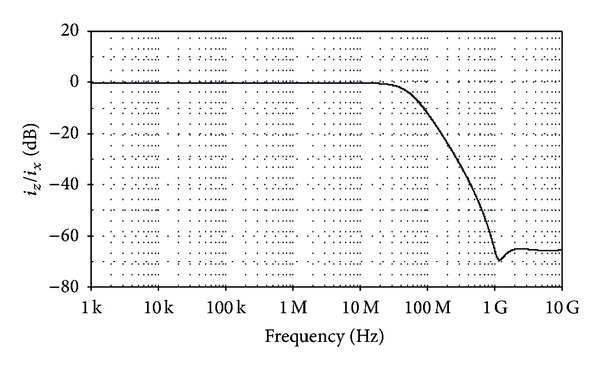
Simulated frequency response of the current transfer *i*_*z*_/*i*_*x*_ characteristic.

In Figure 3, the theoretical and simulated values of the parasitic resistance *R*_*x*_ of the proposed CCCTA against the biasing current *I*_*A*_ are plotted. In the plots, the biasing current *I*_*A*_ was adjusted from 5 *μ*A to 300 *μ*A. As shown, the simulated results agree well with the theory. Next, the DC current transfer behavior has been investigated. A DC sweep simulation has been performed, to demonstrate the range where the current through *z*-terminal is equal to the one applied to *x*-terminal. The resulting plots when *I*_*A*_ = 50 *μ*A are shown in Figure 4, which can observe that the output offset current at the *z*-terminal was found to be approximately 300 nA.  Figure 5 shows the current transfer characteristic from *x*-terminal to *z*-terminal as a function of frequency. One can measure from the simulation results that the −3 dB bandwidth of the current transfer *i*_*z*_/*i*_*x*_ has a value of about 47 MHz.

**Figure 6:**
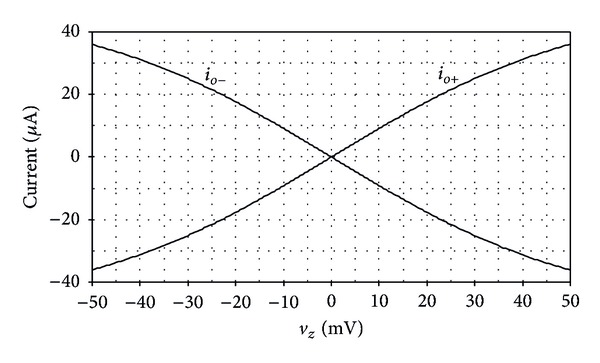
Simulated DC voltage-to-current transfer characteristic between *v*_*z*_ and *i*_*o*±_.

**Figure 7:**
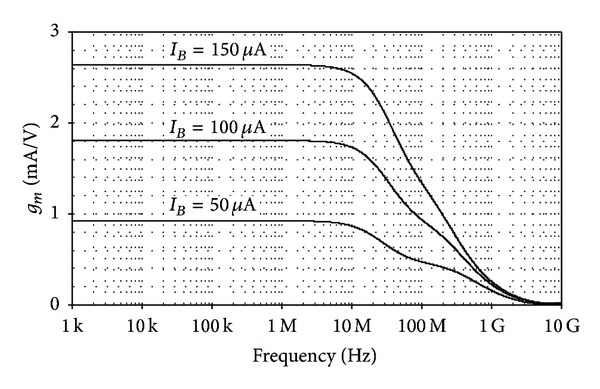
Simulated frequency responses of the transconductance gain *g*_*m*_ characteristic.

Figure 6 shows the plots of both output currents *i*_*o*+_ and *i*_*o*−_ against the input voltage *v*_*z*_. It can be measured from the plots that the voltage *v*_*z*_ linearly converts into output signal currents *i*_*o*+_ and *i*_*o*−_ with nonlinearity of less than 1% for the input voltage range of −50 mV to 50 mV. The simulated frequency responses of the transconductance gain *g*_*m*_ characteristic for three different values of *I*_*B*_, that is, *I*_*B*_ = 50 *μ*A, 100 *μ*A, and 150 *μ*A, are depicted in Figure 7. The results indicate that the bandwidth in order of megahertz is achieved. In addition, the total power dissipation of this circuit was found to be <0.13 mW.  Table 1 compares the major performances between the proposed BiCMOS CCCTA of Figure 2 and the previous bipolar CCCTA reported in [6]. They were obtained with the bias currents *I*_*A*_ = 50 *μ*A and *I*_*B*_ = 100 *μ*A.

## 5. Resistorless Analog Function Realization

In order to underline the potential of the proposed CCCTA, two illustrative analog function circuits have been implemented and discussed in the following subsections. The circuits were realized based on the employment of the minimum number components, thereby reducing the total power consumptions. Also, they do not need any additional passive resistor, which result in the integrable circuit design, as well as simple and compact structures.

### 5.1. Current-Mode Universal Filter

**Figure 8:**
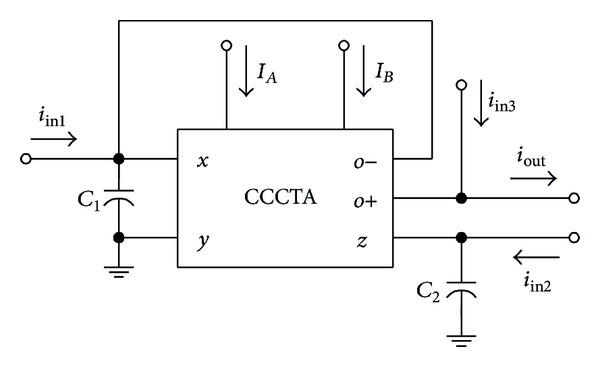
CCCTA-based TISO current-mode universal filter.

As the first application example, the proposed CCCTA was used to realize the resistorless current-mode universal filter. A simple three-input single-output (TISO) filter using a single CCCTA and only two grounded capacitors is described. The configuration is shown in Figure 8. By straightforward analysis, the single-output current function realized by this configuration is found to be

(9) $$\begin{array}{c} I_{\text{out}}\left(s\right)=\frac{D\left(s\right)I_{\text{in}3}-\left(sC_{1}R_{x}+1\right)g_{m}I_{\text{in}2}+g_{m}I_{\text{in}1}}{D\left(s\right)}, \end{array}$$

where

(10) $$\begin{array}{c} D\left(s\right)=s^{2}R_{x}C_{1}C_{2}+sC_{2}+g_{m}. \end{array}$$

From the above expressions, the following can be summarized.

1. The LP response is obtained with *I*_in1_ = *I*_in_ (an input current signal) and *I*_in2_ = *I*_in3_ = 0.
2. The BP response is obtained with *I*_in1_ = *I*_in2_ = *I*_in_ and *I*_in3_ = 0.
3. The HP response is obtained with *I*_in2_ = *I*_in3_ = *I*_in_, *I*_in1_ = 0, and *C*_2_ = *g*_*m*_ *R*_*x*_ *C*_1_.
4. The BS (bandstop) response is obtained with *I*_in1_ = *I*_in2_ = *I*_in3_ = *I*_in_ and *C*_2_ = *g*_*m*_ *R*_*x*_ *C*_1_.
5. The AP (all pass) response is obtained with *I*_in1_ = *I*_in2_ = *I*_in3_ = *I*_in_ and *C*_2_ = 2*g*_*m*_ *R*_*x*_ *C*_1_.

Clearly, the configuration of Figure 8 can be used as a three-input single-input current-mode universal filter that can realize all the five standard types of the biquad filter functions. Also from (9) and (10), the natural angular frequency (*ω*_*o*_) and bandwidth (BW) of the filter in all cases are given, respectively, by

(11) $$\begin{array}{c} \omega_{o}=\sqrt{\frac{g_{m}}{R_{x}C_{1}C_{2}}},\\ \text{BW}=\frac{1}{R_{x}C_{1}}. \end{array}$$

It can be observed that we can tune the values of the filter parameters *ω*_*o*_ and BW by controlling *g*_*m*_ and/or *R*_*x*_.

**Figure 9:**
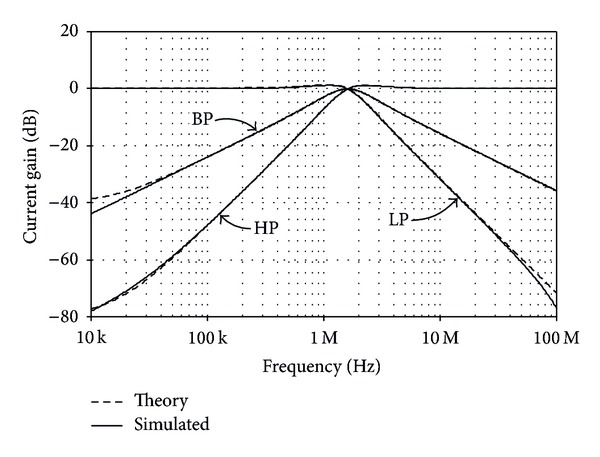
Theory and simulated LP, BP, and HP current responses for the universal filter of Figure 8.

**Figure 10:**
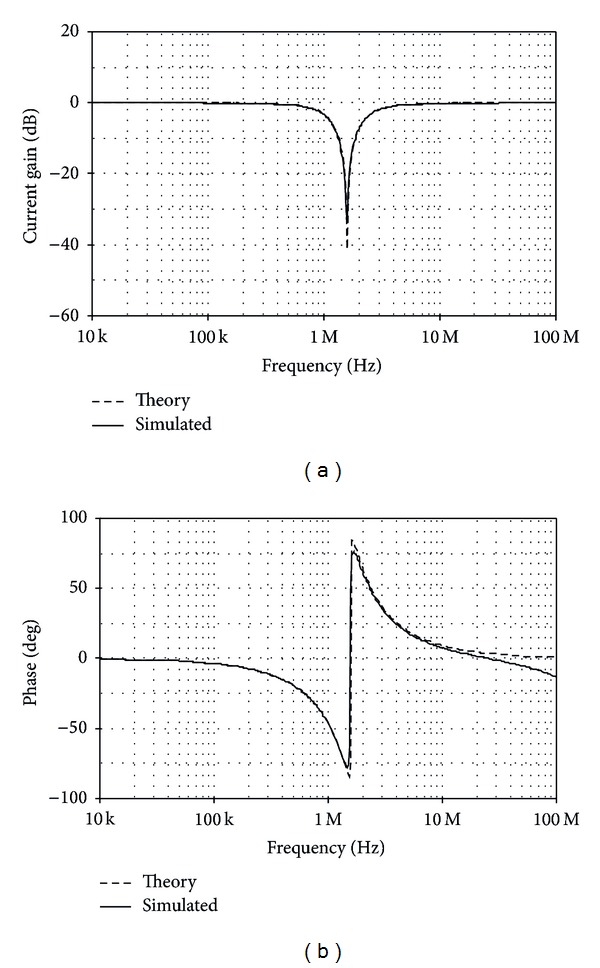
Theory and simulated BS frequency characteristics for the filter of Figure 8: (a) gain responses and (b) phase responses.

**Figure 11:**
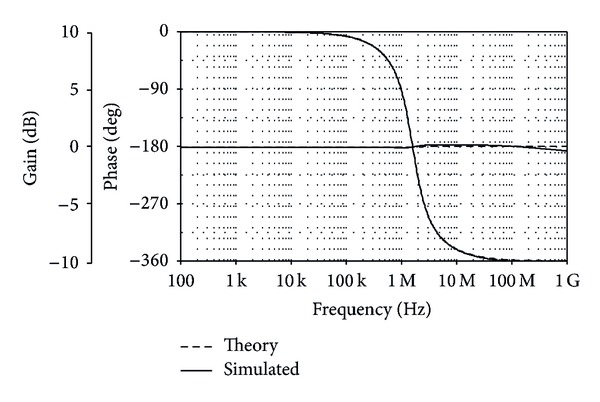
Theory and simulated AP frequency characteristics for the filter of Figure 8.

The TISO resistorless current-mode universal filter of Figure 8 was also simulated. The active and passive component values have been chosen as *I*_*A*_ = *I*_*B*_ = 50 *μ*A and *C*_1_ = *C*_2_ = 100 pF, to obtain the filter responses with a natural angular frequency of *f*_*o*_ = *ω*_*o*_/2*π*≅1.53 MHz. The theory and simulated frequency characteristics for LP, BP, HP, and BS are shown in Figures 9 and 10, respectively.  Figure 11 also shows the AP frequency characteristics of the filter in Figure 8 when *C*_1_ = 50 pF and *C*_2_ = 100 pF.

### 5.2. Floating Inductance Simulator

**Figure 12:**
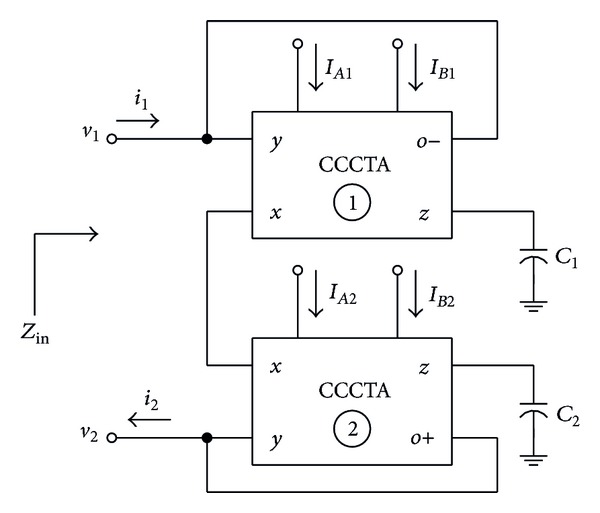
CCCTA-based floating inductance simulator circuit.

Figure 12 represents the lossless floating inductance simulator circuit consisting of two CCCTAs and two grounded capacitors. Taking *g*_*m*_ = *g*_*m*1_ = *g*_*m*2_ and *C* = *C*_1_ = *C*_2_, the input impedance of the simulator is obtained as

(12) $$\begin{array}{c} Z_{\text{in}}=sL_{\text{eq}}≅\frac{s\left(R_{x1}+R_{x2}\right)C}{g_{m}}, \end{array}$$

where *R*_*xi*_ is the parasitic resistance *R*_*x*_ of the *i*th CCCTA (*i* = 1,2). It is obvious that the realized equivalent inductance value is found to be *L*_eq_ = (*R*_*x*1_ + *R*_*x*2_)*C*/*g*_*m*_, which is electronically controllable by adjusting *R*_*xi*_ and/or *g*_*m*_. Additionally, if *v*_2_ = 0, a grounded inductance simulator can also be realized from the configuration of Figure 12.

**Figure 13:**
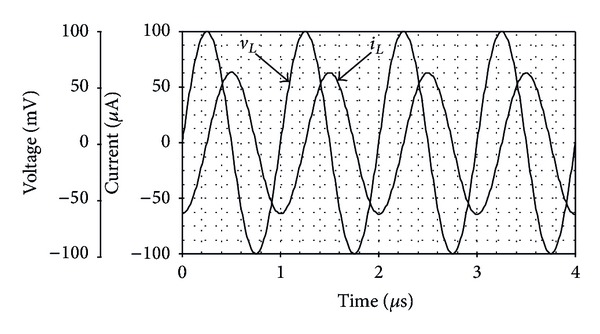
Simulated voltage and current waveforms for the inductance simulator of Figure 12.

**Figure 14:**
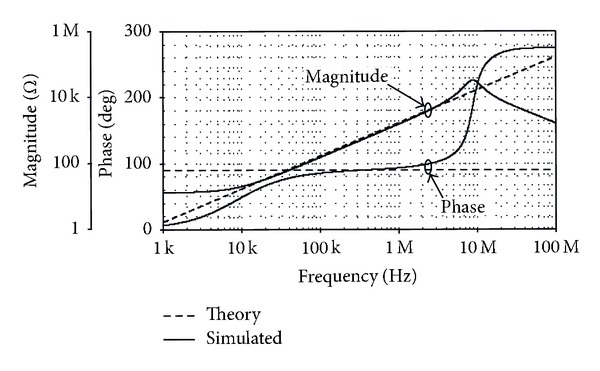
Theory and simulated frequency responses of the inductance simulator in Figure 12.

**Figure 15:**
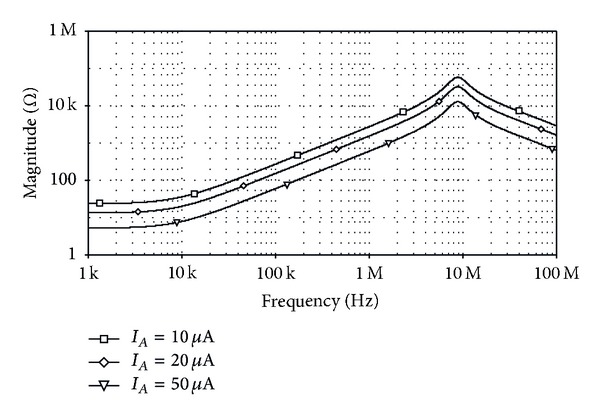
Simulated magnitude responses of the inductance simulator in Figure 12 by varying *I*_*A*_.

To verify the performance of the derived inductance simulator of Figure 12, the circuit was simulated and compared with the ideal inductor. For this purpose, the following component values were taken as *R*_*x*1_ = *R*_*x*2_ = 2.6 kΩ (*I*_*A*1_ = *I*_*A*2_≅20 *μ*A), *g*_*m*1_ = *g*_*m*2_ = 0.96 mA/V (*I*_*B*1_ = *I*_*B*2_≅50 *μ*A), and *C* = *C*_1_ = *C*_2_ = 50 pF, which results in *L*_eq_≅0.27 mH. The simulated voltage and current waveforms of the floating inductance simulator circuit of Figure 12 when a 1 MHz sinusoidal signal is applied are shown in Figure 13. From the results, the phase shift between the current and voltage is about 93°, which is in close correspondence with the expected value equal to 90°. Further, the frequency-dependent impedance of the simulator is shown in Figure 14. It may be noted that the simulator operates correctly along the frequency range 10 kHz to 4 MHz.  Figure 15 also shows the frequency characteristics of the inductance simulator for three different values of *R*_*x*_, where *R*_*x*_ = *R*_*x*1_ = *R*_*x*2_  (*I*_*A*_ = *I*_*A*1_ = *I*_*A*2_). The simulations were performed by varying *R*_*x*_ = 5.2 kΩ (*I*_*A*_≅10 *μ*A), *R*_*x*_ = 2.6 kΩ (*I*_*A*_≅20 *μ*A), and *R*_*x*_ = 1.04 kΩ (*I*_*A*_≅50 *μ*A), to obtain *L*_eq_≅0.54 mH, 0.27 mH, and 0.108 mH, respectively.

## 6. Closing Remarks

In this paper, a simplified structure of the current-controlled conveyor transconductance amplifier (CCCTA) in BiCMOS technology has been introduced and characterized. The circuit is capable of operating at ±1 V supply voltages and can operate to a frequency of about 40 MHz. The proposed CCCTA is implemented with standard 0.35 *μ*m BiCMOS real process parameters. Some resistorless circuit implementations with minimum component count and the added advantage of electronic tuning property realizing from the proposed CCCTA are also given. The simulation results have been performed for the designed CCCTA and its applications to verify the theoretical analysis.

## Figures and Tables

**Table 1:** Main performances of the CCCTA reported in [6] and the proposed one of Figure 2, for *I*_*A*_ = 50 *μ*A and *I*_*B*_ = 100 *μ*A.

Parameters	CCCTA of [6]	Proposed CCCTA of Figure 2
Technology	ALA400 bipolar	0.35 *μ*m BiCMOS
Supply voltages	±1.5 V	±1 V
Power dissipation	1.48 mW	0.13 mW
−3 dB bandwidth for *v*_*x*_/*v*_*y*_	105 MHz	82 MHz
−3 dB bandwidth for *i*_*z*_/*i*_*x*_	34 MHz	47 MHz
−3 dB bandwidth for *i*_*o*±_/*v*_*z*_	30 MHz	40 MHz
*R*_*x*_	260 Ω (@*I*_*B*1_ = 50 *μ*A)	1.04 kΩ (@*I*_*A*_ = 50 *μ*A)
*g*_*m*_	0.95 mA/V–2.78 mA/V (@*I*_*B*2_ = 50 *μ*A–150 *μ*A)	0.925 mA/V–2.64 mA/V (@*I*_*B*_ = 50 *μ*A–150 *μ*A)
Parasitic resistance at port *y* (*R*_*y*_)	7.24 MΩ	262 kΩ
Parasitic resistance at port *z* (*R*_*z*_)	123.26 kΩ	80 kΩ
Parasitic resistance at port *o*± (*R*_*o*±_)	207.87 kΩ	740 kΩ
